# Supplementary material for: Partitioning surface ligands on nanocrystals for maximal solubility
Source: Nat Commun. 2019 Jun 5;10:2454. doi: 10.1038/s41467-019-10389-5 (PMC6549164; doi:10.1038/s41467-019-10389-5)
Supplement: Supplementary file 1 — Supplementary Information [file 41467_2019_10389_MOESM1_ESM.pdf]

Supplementary Information

Partitioning surface ligands on nanocrystals for maximal solubility

Xueqian Kong, Xiaogang Peng et al.

Supplementary Methods

Chemicals. Cadmium oxide (CdO, 99.998%), myristic acid (98%), stearic acid (HSt, >90%), selenium power (200 mesh, 99.999%), 1-octadecene (ODE, 90%) and hexanoic acid (98%) were purchased from Alfa-Aesar. All organic solvents were obtained from Sinopharm Reagents. All chemicals were used directly without further purification.

Synthesis of CdSe-ligands complexes. The synthesis of CdSe nanocrystals was performed by injecting a 1.0 mL Se-octadecene suspension (0.2 mol L^{-1})[1] into a hot (250 °C) mixture of CdO (0.0514 g, 0.4 mmol), myristic acid (0.1142 g, 0.5 mmol) and 6.3 g of ODE in a 50 mL three-neck flask. Needle-tip aliquots were taken for UV–vis and photoluminescence (PL) measurements to monitor the reaction. When the targeted size of nanocrystals was achieved (i.e. when the lowest-energy absorption peak in UV-vis spectra reached 552 nm), the reaction mixture was cooled to room temperature for purification.

Purification of CdSe-ligands complexes. The reaction mixture of the CdSe-ligands complexes (1 mL) was loaded into a 4 mL vial and kept at 50 °C as a clear solution. Into the vial, a mixture of acetone, chloroform and methanol (volume ratio 1:1:1, 2 - 3 mL in total) was added. The vial was centrifuged for 60 s at the temperature of 50 °C. The supernatant was removed quickly. The precipitate was dissolved in 0.5 mL toluene, and then the mixture of acetone, chloroform and methanol was added again to start the second cycle of precipitation, centrifugation and decantation. In the third step, the precipitate was dissolved in 0.5 mL chloroform and further precipitated by adding 0.3 mL acetonitrile with additional centrifugation and decantation. The chloroform-acetonitrile procedure was repeated once. Finally, the sample was vacuumed for 12 hours to remove residual solvents.[2,3] It has been verified by infrared (IR) measurement that remaining ODE and free

acids had been fully removed after the third step (Supplementary Fig. 1c).

Preparation of nanocrystal-ligands complexes with mixed ligands. 10 mg purified nanocrystal-ligands complexes with pure myristate ligands were dissolved in 0.5 mL chloroform in a 4 mL vial and kept at 50 °C as a clear solution. Hexanoic acid with molar ratios ranging from 0.1 to 2 relative to bonded myristate ligands was added into solution for 2 hours. Then chloroform-acetonitrile purification procedure was repeated once or twice. Finally, the samples were vacuumed for 12 hours to remove residual solvents.

General characterizations. Transmission electron microscopy (TEM) images were taken on a Hitachi 7700 transmission electron microscope operating at 100 kV, and the nanocrystals were deposited onto ultrathin carbon films supported by copper grid. The sizes of CdSe nanocrystals appeared uniform for samples with variable hexanoate fractions less than 0.90 (Supplementary Fig. 1a). When f_{He} was close to or higher than 0.90, some coagulation could be observed, which might suggest the complexes were compromised by the excess hexanoic acids. Therefore, samples with high hexanoate fractions have not been considered in our study.

The IR spectra of nanocrystal-ligands complexes with mixed ligands (performed on a Thermo Scientific Nicolet 380 spectrometer) showed some weakening of peaks in the isomeric region (1200 – 1400 cm^{-1}) compared to those of nanocrystal-ligands complexes with pure ligands (Supplementary Fig. 1d). The IR result suggests a loosened packing of hydrocarbon chains for nanocrystal-ligands complexes with mixed ligands which is consistent with the solid-state NMR studies described in the main text.

The UV-vis spectra (collected in chloroform solution on a Shimadzu UV-2600 spectrometer) showed that the lowest-energy absorption peaks of all nanocrystal-ligands complexes with mixed

ligands remained around the 552 nm which confirmed that the size of nanocrystals did not change after ligand exchange (Supplementary Fig. 1e).

Small-angle X-ray scattering (SAXS) measurements were performed on a Rigaku D/Max 2550/PC diffractometer utilizing Cu K_{α} radiation. For the $f_{He} = 0.68$ sample, the peak shifted to a larger q compared to that of $f_{He} = 0$ sample suggesting a smaller spacing between particles.

Measurement of ligand fractions. The fraction of hexanoate on nanocrystal-ligands complexes with mixed ligands (Supplementary Table 1) was determined by gas chromatography (GC) Shimadzu GC-2014C. 10 mg nanocrystal-ligands complexes with mixed ligands were fully dissolved in 1 ml hexane, and 20 μl saturated hydrochloric acid was added to digest complexes under argon environment. The molar ratio of hexanoic acid to myristic acid in the digested solution is assumed to be the same as the ratio of those two ligands on nanocrystal surface. The GC working curves of myristic acid and hexanoic acid were determined by the mixed solutions of those two acids with concentration range from 1 to 100 μM.

Surface density of ligands. The surface density of nanocrystal-ligands complexes with pure ligands is determined to be ~ 135 ligands per crystal based on the carbon mass fraction measured by elemental analysis[4] . The chemical formula for nanocrystal-ligands complex with pure ligand was assumed to be $(CdSe)_m(CdMy_2)_x$. Here, m equals to 229 for spherical CdSe nanocrystals with a diameter of 3.0 nm, and x was determined by carbon mass fraction.

$$\text{carbon mass fraction} = \frac{2x \cdot 14M_{\mathrm{C}}}{229 \times (M_{\mathrm{Cd}} + M_{\mathrm{Se}}) + x \cdot \left(M_{\mathrm{Cd}} + 2 \times M_{\mathrm{My}}\right)} \tag{1}$$

Where M_{C}, M_{Cd}, M_{Se} and M_{My} are the molar mass of carbon, cadmium, selenium and myristate.

Since the molar mass of CdSe core does not change after ligand exchange, the surface density of ligand n of samples with various known exchanged ratio can be determined.

$$\text{carbon mass fraction} = \frac{(14C_{\text{My}} + 6C_{\text{He}})M_{\text{c}}n}{M_{\text{Core}} + (M_{\text{My}}C_{\text{My}} + M_{\text{He}}C_{\text{He}})n} \quad (2)$$

C_{My} and C_{He} are the molar fraction of myristate and hexanoate on nanocrystals determined by GC. M_{c}, M_{He}, M_{My} and M_{Core} are the molar mass of carbon atom, hexanoate, myristate and the core of CdSe nanocrystals, respectively. The carbon mass fractions were determined by elemental analysis (using Elementar Vario MICRO) of purified samples. M_{Core} equals to 5.1×10^4 g mol^{-1} for nanocrystals of 3.0 nm in diameter. The general formula for nanocrystal-ligands complexes with mixed ligands is $(CdSe)_m(CdMy_2)_x(CdHe_2)_y$. The values of n for samples with various hexanoate fractions is shown in Supplementary Table 1.

Solubility measurement. The solubility at room temperature was determined by the UV-vis absorbance of saturated solutions of CdSe complexes. The concentration of supernatant equals to the solubility[5].

A different procedure to measure solubility of complexes at various temperatures was performed by the scattering method[4,6,7]. A known concentration of dissolved nanocrystal-ligands complexes in chloroform was slowly cooled down from a relatively high temperature. The scattering intensity of the solution for a 750 nm laser was recorded during the cooling process. The scattering intensity shows a sudden jump when the concentration reaches the solubility at the specific temperature. The experimental data (Supplementary Fig. 1g) were plotted and fitted according to the equation described in Ref. 5 and 6 to get the molar dissolution enthalpy $\Delta^m H_{\text{NC}}$ and molar fusion entropy $\Delta^{\text{m}} S_{\text{NC}}$.

$$\frac{1}{T} = -\frac{R}{\Delta^m H_{\text{NC}}} ln\chi + \frac{\Delta^m S_{\text{NC}}}{\Delta^m H_{\text{NC}}} \quad (3)$$

Center-band only detection exchange (CODEX). The pulse sequence for the ^{13}C CODEX experiments is shown in Supplementary Fig. 2a[8,9]. After ^{1}H-^{13}C cross polarization (CP), the first

rotor-synchronized π-pulses train recouples the chemical shift anisotropy (CSA) interaction of ^{13}C under MAS with ^{1}H decoupling. During the mixing time (τ_m), ^{13}C spin diffusion and/or molecular reorientation change the chemical shift frequency and prevent complete signal recovery of the second rotor synchronized π-pulses recoupling train. A z-filter (τ_z) is added after the second π-pulses train to correct for spin-lattice relaxation (T_1) effects during variable τ_m. Two experiments, an exchange experiment (S) with the desired τ_m and a short τ_z (1 ms), and a reference experiment (S_0) with interchanged τ_z and τ_z, were performed. The normalized intensity, S/S_0, was measured as a function of the mixing time until it reached a plateau[10]. We confirmed that the isolated carboxylate groups do not alter CODEX decay due to the rigidity of themselves (Supplementary Fig. 2c). Therefore, the measured CODEX decays were purely modulated by the ^{13}C-^{13}C coupling arising from the neighboring ^{13}C-labelled ligands.

Analysis of CODEX curves and ligand partition. For spin diffusion among n spins, the time-evolution of the n-dimensional vector, **M**(t), describing the z magnetization, is given by

$$\frac{\mathrm{d}\mathbf{M}(t)}{\mathrm{d}t} = -\hat{\mathbf{K}}\mathbf{M}(t) \tag{4}$$

$$\frac{\mathbf{M}(t)}{\mathbf{M}(0)} = \mathrm{e}^{-\hat{\mathbf{K}}t} \tag{5}$$

where $\hat{\mathbf{K}}$ is the n-dimensional exchange matrix consisting of the rate constants, k_{ij}, between two spins. Detailed balance of equilibrium magnetization requires that the sum of each column of the $\hat{\mathbf{K}}$ matrix be zero and that the rate constants satisfy $k_{ij} = k_{ji}$ for equal populations of equilibrium magnetization.[11] The $\hat{\mathbf{K}}$ matrix for n spins is

$$\{K\}_{ij} = (\delta_{ij} - 1)k_{ij} + \delta_{ij}\sum_{m\neq j}^{n} k_{mj} \tag{6}$$

The rate constants k_{ij} is

$$k_{ij} = \frac{\pi}{2}\omega_{ij}^2 F_{ij}(0) = \frac{a}{r_{ij}^6} F_{ij}(0) \quad (7)$$

with $a = 7.1565 \times 10^8 \text{Å}^6\text{s}^{-2}$ for ^{13}C considering powder average.[12] ω_{ij} is the homonuclear dipolar coupling which is proportional to $1/r_{ij}^6$, where r_{ij} is the distance between two spins. $F_{ij}(0)$ is the overlap integral and for singly labeled system the overlap integral is approximated as the same for all spin pairs.[13] The range of $F(0)$ is determined by model compounds, 1-^{13}C-glycine, 1-^{13}C-leucine and 1-^{13}C-phenylalanine whose $F(0)$ are 32 μs, 4 μs and 25 μs respectively Supplementary Fig. 2b). The $F(0)$ used in our nanocrystal-ligands complexes is 20 μs which is in the reasonable range of model compounds.

Generating ligand partition models. The initial distribution of pure ligands was generated by a numerical iteration method. A fixed number of sites were first randomly distributed on the surface of a sphere. We implemented a hypothetical interaction force between two sites which is proportional to $1/r^2$ where r is the distance between two sites. The sites were allowed to redistribute on the spherical surface following the tangential direction of the total force vector. The uniform distribution was obtained when the resultant force equaled zero for all sites. The non-uniform distributions were obtained from the uniform distribution by generating bundles with different numbers of ligands around a center point (Supplementary Fig. 2e,f). Both square-like bundles and diamond-like bundles give similar results. The CODEX decay is more relevant to the average inter-ligand distances rather the actual arrangement. The inter-ligand distance for nearest neighbors in a bundle is set at 0.39 nm since it gives the best fit to the experimental data.

The ligand partitions of nanocrystal-ligands complexes with mixed ligands were generated from the non-uniform distribution with 5 ligands in one bundle as which matched the ligand distribution on nanocrystal-ligands complexes with pure ligands. The random partition was

generated by randomly removing individual sites. The areal partition was generated by randomly removing the bundles.

Analysis of deuterium lineshapes and dynamic modes. The ^{2}H lineshape of a static CD_2 group is a Pake pattern with a C_Q of 167 kHz. In the intermediate regime, the motional-averaged pattern with a quadrupolar splitting of 40 kHz indicates a dynamic process of trans-gauche^{+}-gauche^{-} (tgg) rotation[14]. Such tgg rotation is equivalent to a 3-site jump on a cone which is common for chains with mostly trans conformations. The sharp pattern at higher temperatures indicates a more isotropic motion with larger amplitude. We define such motion as the cone diffusion mode which is similar to dynamics of the terminal part of a polyethylene chain in the amorphous region[15]. The cone diffusion mode can be considered as a wobbling motion superimposed on tgg rotation. This superimposed cone diffusion model fits the sharp ^{2}H peaks rather than a simple cone diffusion without tgg rotation (Supplementary Fig. 3c). Experimental deuterium lineshapes were fitted by the superposition of simulated patterns for different motional modes: static, tgg rotation and cone diffusion. The simulations were performed with the sola module of Topspin 3.5pl7 and EXPRESS package[16].

Dipolar coupling chemical shift correlation (DIPSHIFT). DIPSHIFT pulse sequence is shown in Supplementary Fig. 5a[17]. After ^{1}H-^{13}C CP, ^{1}H homonuclear decoupling (frequency-switched Lee-Goldburg, FSLG)[18-20] is applied and the natural abundant ^{13}C signal evolves under ^{1}H-^{13}C dipolar coupling and chemical shift anisotropy (CSA). The π-pulse between two rotor periods refocused the CSA, and therefore the ^{13}C evolution with pure ^{1}H-^{13}C dipolar coupling is acquired.

DIPSHIFT works with different motional regimes, including intermediate ($10^{-6} < \tau_c < 10^{-3}$ s) and fast ($\tau_c < 10^{-6}$ s) motions[21]. For rigid molecules, the evolution of ^{1}H-^{13}C dipolar

coupling is symmetrical with respect to the half rotor period. When molecules are in intermediate regime i.e. the rate of molecular motion and ^{1}H-^{13}C dipolar coupling are comparable, the evolution curve loses its symmetry. But when the motion rate exceeds the dipolar coupling, it shows a symmetrical curve with a shallower dip indicating a reduced (averaged) coupling constant. The DIPSHIFT curves of our samples were symmetric, which indicated that the ligands were in the fast motion regime.

Calculating opening angles of cone diffusion. The DIPSHIFT curves of a CH_2 group under fast motion can be calculated as:

$$I(t) = \mathrm{Tr}(\boldsymbol{\rho}(t)\mathbf{S}_+) \tag{8}$$

$$\boldsymbol{\rho}(t) = \mathrm{e}^{-\mathrm{i}\int_0^t \mathbf{H}(t)dt}\boldsymbol{\rho}(0)\mathrm{e}^{\mathrm{i}\int_0^t \mathbf{H}(t)dt} \tag{9}$$

Where $I(t)$ is the signal intensity in time domain and $\mathbf{S}_+$ is the observation operator. $\boldsymbol{\rho}(t)$ is the density matrix of the observed spin and $\boldsymbol{\rho}(0)$ is the initial density matrix at t = 0. To account for the CH_2 three-spin system, $\mathbf{S}_+$ and $\boldsymbol{\rho}(0)$ need to be expanded into eighth order matrices.

$$\mathbf{S}_+ = \mathbf{E} \otimes \mathbf{E} \otimes \mathbf{S}_+^c \tag{10}$$

$$\boldsymbol{\rho}(0) = \mathbf{E} \otimes \mathbf{E} \otimes \mathbf{S}_\mathrm{x}^c \tag{11}$$

where **E** is the second order unit matrix. **H**(t) is the Hamiltonian of a CH_2 spin system which includes dipolar coupling between the observed ^{13}C spin and the other two ^{1}H spins.

$$\mathbf{H}(\boldsymbol{\Omega}, t) = \omega_\mathrm{d}^\mathrm{LAB}(\boldsymbol{\Omega}_1, t)\mathbf{I}_{1\mathrm{z}}\mathbf{S}_\mathrm{z} + \omega_\mathrm{d}^\mathrm{LAB}(\boldsymbol{\Omega}_2, t)\mathbf{I}_{2\mathrm{z}}\mathbf{S}_\mathrm{z} \tag{12}$$

$$\mathbf{I}_{1\mathrm{z}}\mathbf{S}_\mathrm{z} = \mathbf{I}_{1\mathbf{z}}^\mathrm{H} \otimes \mathbf{E} \otimes \mathbf{S}_\mathbf{z}^\mathrm{C} \tag{13}$$

$$\mathbf{I}_{2\mathrm{z}}\mathbf{S}_\mathrm{z} = \mathbf{E} \otimes \mathbf{I}_{2\mathbf{z}}^\mathrm{H} \otimes \mathbf{S}_\mathbf{z}^\mathrm{C} \tag{14}$$

$\omega_\mathrm{d}^\mathrm{LAB}(\boldsymbol{\Omega}_i, t)\mathbf{I}_{i\mathrm{z}}\mathbf{S}_\mathrm{z}$ is the dipolar coupling between the i th I spin (^{1}H) and S spin (^{13}C) and $\omega_\mathrm{d}^\mathrm{LAB}$ is the coupling constant in laboratory frame which depends on the alignment of C-H vector. $\boldsymbol{\rho}(t)$ can

be calculated as

$$\boldsymbol{\rho}(t)=\begin{pmatrix} 0 & \frac{1}{2}\mathrm{e}^{-\mathrm{i}\frac{D_1+D_2}{2}} & & & & & & \\ \frac{1}{2}\mathrm{e}^{\mathrm{i}\frac{D_1+D_2}{2}} & 0 & & & & & 0 & \\ & & 0 & \frac{1}{2}\mathrm{e}^{-\mathrm{i}\frac{D_1-D_2}{2}} & & & & \\ & & \frac{1}{2}\mathrm{e}^{\mathrm{i}\frac{D_1-D_2}{2}} & 0 & & & & \\ & & & & 0 & \frac{1}{2}\mathrm{e}^{\mathrm{i}\frac{D_1-D_2}{2}} & & \\ & & & & \frac{1}{2}\mathrm{e}^{-\mathrm{i}\frac{D_1-D_2}{2}} & 0 & & \\ & 0 & & & & & 0 & \frac{1}{2}\mathrm{e}^{\mathrm{i}\frac{D_1+D_2}{2}} \\ & & & & & & \frac{1}{2}\mathrm{e}^{-\mathrm{i}\frac{D_1+D_2}{2}} & 0 \end{pmatrix} \quad (15)$$

where $D_i(t)=\int_0^t \omega_{\mathrm{d}}^{\mathrm{LAB}}(\boldsymbol{\Omega}_i,t)dt,\ (i=1,2)$ with $D_i(0)=0$. Then the signal intensity can be calculated as:

$$I(t)=\cos(D_1+D_2)+\cos(D_1-D_2) \quad (16)$$

$$I(0)=2$$

So, the final DIPSHIFT intensity equals to

$$\frac{I(t)}{I(0)}=\frac{cos(D_1+D_2)+cos(D_1-D_2)}{2} \quad (17)$$

For heteronuclear dipolar coupling, the coupling constant in lab frame ω_d^{LAB} can be converted from that in principal axis frame ω_d^{PAF} by

$$\omega_{\mathrm{d}}^{\mathrm{LAB}}=\omega_{\mathrm{d}}^{\mathrm{PAF}}\sum_{m,m'=-2}^{2} D_{0m'}^{2}(\boldsymbol{\Omega}_{PC})D_{m'm}^{2}(\boldsymbol{\Omega}_{CR})D_{m0}^{2}(\boldsymbol{\Omega}_{RL}) \quad (18)$$

where D is wigner rotation matrix, and Ω_{PC}, $\boldsymbol{\Omega}_{\mathrm{CR}}$ and $\boldsymbol{\Omega}_{\mathrm{RL}}$ are the Euler angles (ZYZ) from principal frame (PAF) to crystal (molecular) frame, crystal frame to rotor frame and rotor frame to laboratory frame, respectively.

$$\omega_{\mathrm{d}}^{\mathrm{PAF}}=-\frac{\mu_0\gamma_I\gamma_S}{4\pi r_{IS}^3}\hbar \quad (19)$$

If we choose a proper crystal frame, we can make the Euler angles from PAF to molecular frame of two C-H vectors in one CH_2 group differ only in sign of β_{PC}.

$$\omega_{\mathrm{d}}^{\mathrm{LAB}}(\pm\beta_{\mathrm{PC}}) = \omega_{\mathrm{d}}^{\mathrm{PAF}}[\left(\frac{1}{6}\cos2\alpha(\cos^2\beta+1) \pm \frac{\sqrt{2}}{3}\cos\alpha\cdot\sin\beta\cdot\cos\beta\right)\cos(2\omega_{\mathrm{MAS}}t+2\gamma) + \left(-\frac{2}{3}\cos\alpha\cdot\sin\alpha\cdot\cos\beta \mp \frac{\sqrt{2}}{3}\sin\alpha\cdot\sin\beta\right)\sin(2\omega_{\mathrm{MAS}}t+2\gamma) + \left(\frac{\sqrt{2}}{3}\cos2\alpha\cdot\cos\beta\cdot\sin\beta \mp \frac{2}{3}\cos\alpha\cdot\cos2\beta\right)\cos(\omega_{\mathrm{MAS}}+\gamma) + \left(-\frac{2\sqrt{2}}{3}\cos\alpha\cdot\sin\alpha\cdot\sin\beta \pm \frac{2}{3}\sin\alpha\cdot\cos\beta\right)\sin(\omega_{MAS}t+\gamma)] \quad (20)$$

in which *α, β, γ* are the Euler angles from molecular frame to rotor frame. ω_{MAS} is magic angle spinning rate. After powder averaging, we can get the simulated DIPSHIFT curves. Because we use FLSG for ^{1}H-^{1}H homonuclear decoupling, it generates a scaling factor of $\frac{1}{\sqrt{3}}$ to the measured ^{1}H-^{13}C dipolar coupling.

For quantitative analysis we use dynamic order parameter S[22,23], which is defined as

$$S = \frac{D_{\mathrm{average}}}{D_0} \quad (21)$$

where $\mathrm{D_{average}}$ is the averaged dipolar coupling constant calculated from DIPSHIFT curves and $\mathrm{D_0}$ is the dipolar coupling constant of a rigid molecule.

In cone diffusion model (Supplementary Fig. 5c), order parameters can be converted into the opening angle θ of the cone[24,25] according to

$$S^2 = \left[\frac{\cos\theta(1+\cos\theta)}{2}\right]^2 \quad (22)$$

The DIPSHIFT results of nanocrystal-ligands complexes with pure ligands and mixed ligands are listed in Supplementary Table 2.

Prediction of interaction energy and solubility.

Calculation of ligand-ligand interaction energy

The calculation of ligand-ligand interaction energy was grounded on the following propositions: (1) The geometric confinement of individual ligand implies the energy barrier exerted by their surrounding environment, and therefore it accounts for both intra- and inter-particle ligand

interactions. (2) The inter-atomic distance is determined by the free volume of each methylene unit. (3) The free volume is estimated by the opening angles of methylene units and their respective positions along the chain.

Based on the simplified dispersion interaction model[26], Van der Waals interaction energy between two molecular sites can be expressed as:

$$U = \frac{3\alpha_0^2 h\nu}{4(4\pi\epsilon_0)^2}\frac{1}{|\mathbf{r_1} - \mathbf{r_2}|^6} \tag{23}$$

where U is the interaction energy, $\mathbf{r_1}$ and $\mathbf{r_2}$ are the position vectors of these two sites. For CH_2 groups, the polarizability $\alpha_0/4\pi\epsilon_0$ is equal to $1.84 \times 10^{-30}\mathrm{m}^3$ and the ionization potential $h\nu$ is equal to 1.67×10^{-18} J. In our work, the CH_2 groups were allowed to move inside a confined domain (i.e. free volume) resulting from a circle potential (Supplementary Fig. 6a).

The equivalent motion-averaged interaction energy is

$$U_i = \frac{3\alpha_0^2 h\nu}{4(4\pi\epsilon_0)^2}\int_{S_i} d\boldsymbol{\tau}\int_{l_i}\frac{1}{|\mathbf{r}(\boldsymbol{\tau}) - \mathbf{r}_l(\mathbf{l})|^6}d\mathbf{l} \tag{24}$$

where l_i is the circle potential restricting the *i* th CH_2 and S_i is the free domain of the *i* th CH_2, $\mathbf{r}_l$ is the position vector on the circle potential, and $\mathbf{r}$ is the position vector of CH_2 in the domain.

The radius of circle potential $r_{0,i}$ for *i* th CH_2 is determined by

$$r_{0,i} = 0.18nm + \sum_{i-1} l_{\mathrm{C-C}} \cdot \sin\alpha_i \tag{25}$$

where 0.18 nm is half of averaged minimum distance between two carboxylate groups obtained from CODEX. $l_{\mathrm{C-C}}$ is the length of single C-C bond which is 0.127 nm. α_i is the opening angle of the i th CH_2 group obtained from DIPSHIFT. The free domain S_i is considered as a concentric circle with a reduced radius

$$r_{\mathrm{S},i} = f_{\mathrm{S}} \cdot r_{0,i} \tag{26}$$

where f_{S} is adjustable parameter and it was set to be 0.33 in our case (Supplementary Fig. 6a).

The chemical shift of No. 5 CH_2 in hexanoate can be distinguished from No. 5 CH_2 in myristate, but the opening angles of those two sites are almost the same at the room temperature (Supplementary Table 2). Therefore, the interaction energies of No. 1 to 5 CH_2 units of hexanoates were regarded the same as those at the same positions of myristates.

Calculation of inter-particle interaction energy

The inter-particle interaction energy between the cores of nanocrystals consists of two contributions: the dipolar interaction between nonlocal dipoles (V^{C}) and the dispersive (Van der Waals) interaction between the cores (V^{vdW})[27].

$$E_{\mathrm{core}} = -(V^{\mathrm{C}} + V^{\mathrm{vdW}}) \tag{27}$$

The dipole moment in CdSe nanocrystals is associated with non-centrosymmetric distribution of charges at the nanocrystal surface (Supplementary Fig. 6b). The nonlocal dipoles were assumed to be two opposite point charges placed on the opposite side of a nanocrystal. The potential between point charges of different nanocrystals are described by the Coulombic potentials.

$$V_{mn}^{\mathrm{C}} = \frac{q_m q_n}{4\pi\epsilon_0\epsilon\, r_{mn}} \tag{28}$$

where r_{mn} is the distance between m th and n th point charge, q_m is the charge of the m th point charge (for our CdSe nanocrystal complexes, $q = \mu/D = 0.341\, q_0$, $\mu = 49$ Debye, $D = 3$ nm, $q_0 = 1.6 \times 10^{-19}$ C), ϵ_0 is vacuum permittivity and ϵ is the dielectric constant ($\epsilon = 2$ for hydrocarbon chains). The dipolar potential for a system with N dipoles is

$$V^{\mathrm{C}} = \frac{1}{2}\sum_{m\neq n}^{2N} V_{mn}^{\mathrm{C}} \tag{29}$$

The Van der Waals potential between two spherical particles is described as

$$V_{ij}^{\mathrm{vdW}} = -\frac{A}{12}\left\{\frac{R}{D_{ij}\left[1+\frac{D_{ij}}{4R}\right]} + \frac{1}{1+\frac{D_{ij}}{R}+\frac{D_{ij}^2}{4R^2}} + 2\ln\left(\frac{D_{ij}\left[1+\frac{D_{ij}}{4R}\right]}{R\left[1+\frac{D_{ij}}{R}+\frac{D_{ij}^2}{4R^2}\right]}\right)\right\} \tag{30}$$

where R is the nanoparticle radius ($R = 1.5$ nm for our system), D_{ij} is the distance of two neighboring nanocrystals obtained from SAXS results. A defines the Hamaker constant ($A = 0.3$ eV for CdSe/hydrocarbon chain/CdSe system). The Van der Waals potential for a system with N dipoles is

$$V^{\mathrm{vdW}} = \frac{1}{2}\sum_{i\neq j}^{N} V_{ij}^{\mathrm{vdW}} \tag{31}$$

The inter-particle interaction energy for a system with N dipoles is

$$E_{\mathrm{core}} = -\frac{1}{2}\sum_{m\neq n}^{2N} V_{mn}^{\mathrm{C}} - \frac{1}{2}\sum_{i\neq j}^{N} V_{ij}^{\mathrm{vdW}} \tag{32}$$

Here all the dipoles are in parallel distribution in a face-center cubic lattice with size of $10 \times 10 \times 10$ (Supplementary Fig. 6c).

Predicting total interaction energy and solubility

The total interaction energy between nanocrystal-ligands complexes consists of ligand-ligand interaction energy (E_{ligand}) and inter-particle interaction energy (E_{core}).

$$E_{\mathrm{tot}} = E_{\mathrm{ligand}} + E_{\mathrm{core}} \tag{33}$$

E_{tot} have been obtained for a series of samples with different hexanoate fractions. When $f_{\mathrm{He}} < 0.68$, the total interaction energy can be fitted with a polynomial Supplementary Fig. 6d).

$$E_{\mathrm{tot}}\ (\mathrm{kJ}) = 26.1 f_{\mathrm{He}}^2 - 54.5 f_{\mathrm{He}} + 304.54 \tag{34}$$

Solubility at room temperature was calculated according to

$$\chi = \mathrm{e}^{-\frac{\Delta^{\mathrm{m}} H_{\mathrm{NC}}}{RT}} \mathrm{e}^{\frac{\Delta^{\mathrm{m}} S_{\mathrm{NC}}}{R}} \tag{35}$$

where $\Delta^{\mathrm{m}}\mathrm{H}_{\mathrm{NC}}$ equals to the total interaction energy E_{tot}, and $\Delta^{\mathrm{m}}\mathrm{S}_{\mathrm{NC}}$ was assumed to be a

constant value of 889 J mol^{-1} K^{-1}.

Data availability. The data that support the findings of this study are available from the corresponding authors upon reasonable request

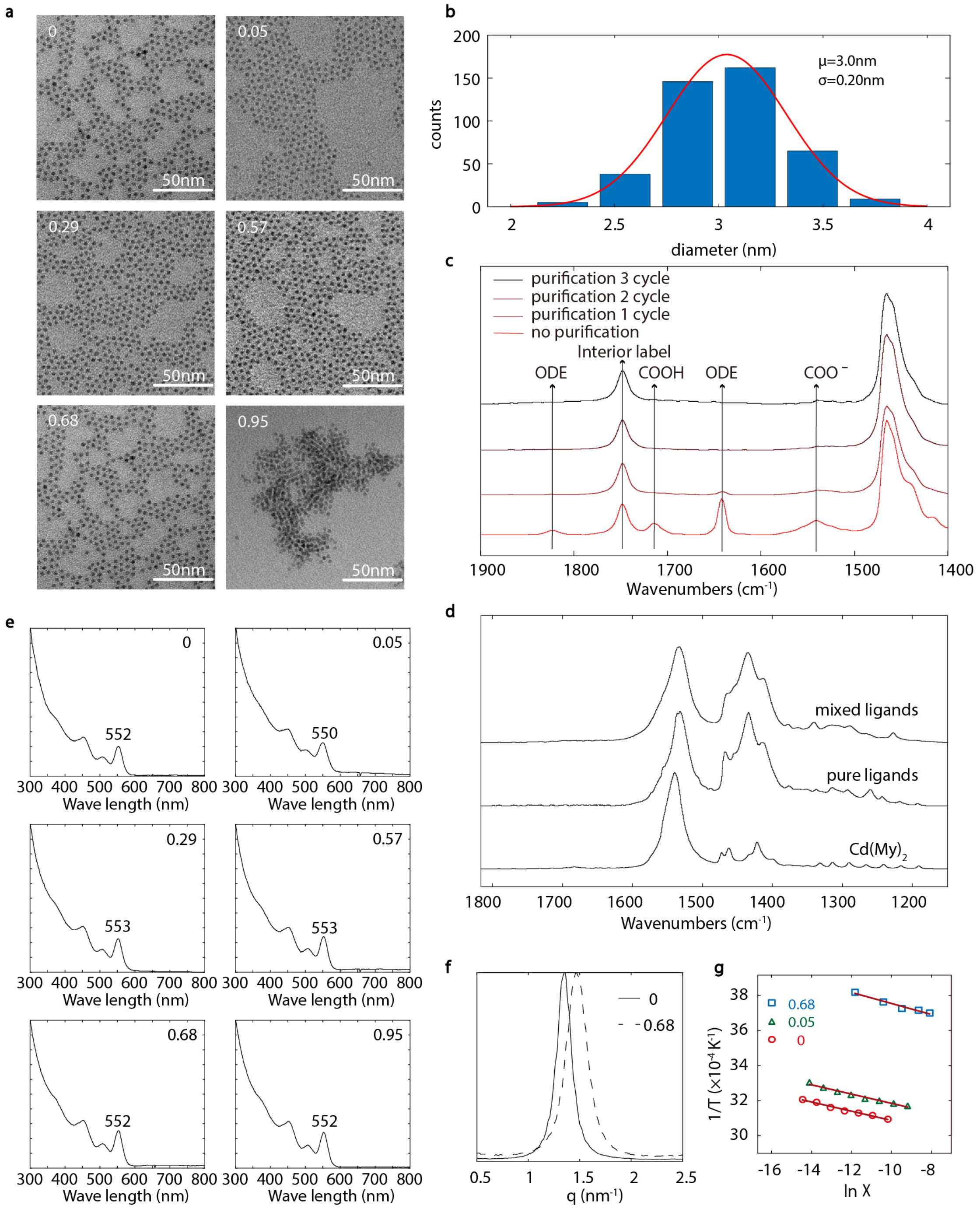

Supplementary Figure 1. Characterization of CdSe nanocrystal-ligands complexes. a, TEM images of samples with different hexanoate fractions (f_{He}) as labeled in each plot. For samples with f_{He} = 0 to 0.68, the nanocrystals are well dispersed. In the sample with f_{He} = 0.95, some of the nanocrystals aggregated as shown in the selected TEM image due to their poor solubility. **b,** Diameter distribution of nanocrystal-ligands complexes with pure ligands getting from the TEM

image. The histogram is fitted by a Gaussian curve whose expectation value (μ) is 3.0 nm and standard deviation (σ) is 0.20 nm. **c,** Infrared spectra of CdSe nanocrystal-ligands complexes solution after different purification cycles. These results indicate successful removal of free ligands, side products, and non-volatile solvents. **d,** Powder infrared spectra of cadmium (II) myristate $Cd(My)_2$, nanocrystal-ligands complexes with pure ligands and nanocrystal-ligands complexes with mixed ligands, indicating carboxylates being the sole ligands of the nanocrystals. **e,** UV-vis spectra for CdSe nanocrystal-ligands complexes measured in solution with different hexanoate fractions. These spectra verified that the inorganic core of the nanocrystal-ligands complexes remained intact upon ligand exchange. **f,** SAXS results of CdSe nanocrystal-ligands complexes with different hexanoate fractions. The shift of the peak is expected due to the shortening of inter-particle distance for complexes with more hexanoates. For nanocrystal-ligands complexes with mixed ligands, the peak width increases significantly, indicating an increase of disorder in the ligand shell. **g,** Solubilities measured with the light scattering method for CdSe nanocrystal-ligands complexes with different hexanoate fractions. The lines are fitting curves using the thermodynamic equation described above.

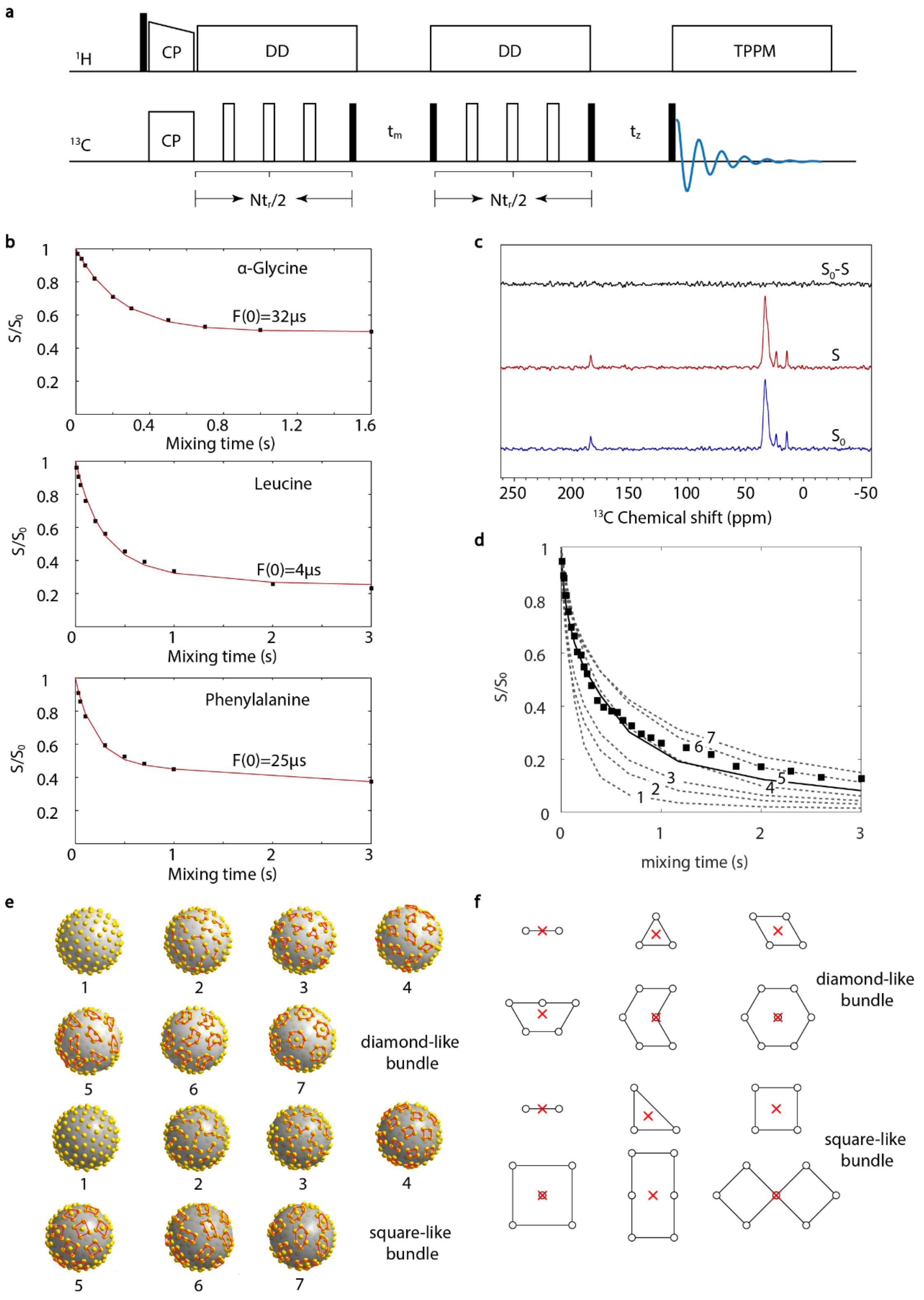

Supplementary Figure 2. CODEX sequence and auxiliary data. a, The ^{13}C CODEX sequence with ^{1}H-^{13}C cross polarization. The first rotor-synchronized π-pulses train recouples the chemical shift anisotropy (CSA) interaction of ^{13}C under MAS. During the mixing time (τ_m), ^{13}C spin

diffusion and/or molecular reorientation change the chemical shift frequency and prevent complete signal recovery by the second rotor synchronized π-pulses recoupling train. **b,** CODEX curves of the carboxylate groups of α-glycine, leucine and phenylalanine with MAS spinning rate of 8 kHz. The best fitting overlap integral, F(0), is shown in the figures. **c,** CODEX results of pure-ligand complexes with unlabeled myristates with natural abundance ^{13}C. The experiment was performed with a mixing time 100 ms and under room temperature. The S and S_0 spectra were identical which confirmed that the molecular reorientation does not alter CODEX curves. **d,** The CODEX decay of nanocrystal-ligands complexes with pure ligands based on the signal of ^{13}C-labeled myristate ligands. The solid and dotted lines are the simulation results of square-like bundle of different sizes. **e,** Schemes of ligand distribution on nanocrystal-ligands complexes with pure ligands with different numbers of ligands in diamond-like bundles and square-like bundles. **f,** Schemes of square-like and diamond-like bundles of different sizes. The red crosses define their center points.

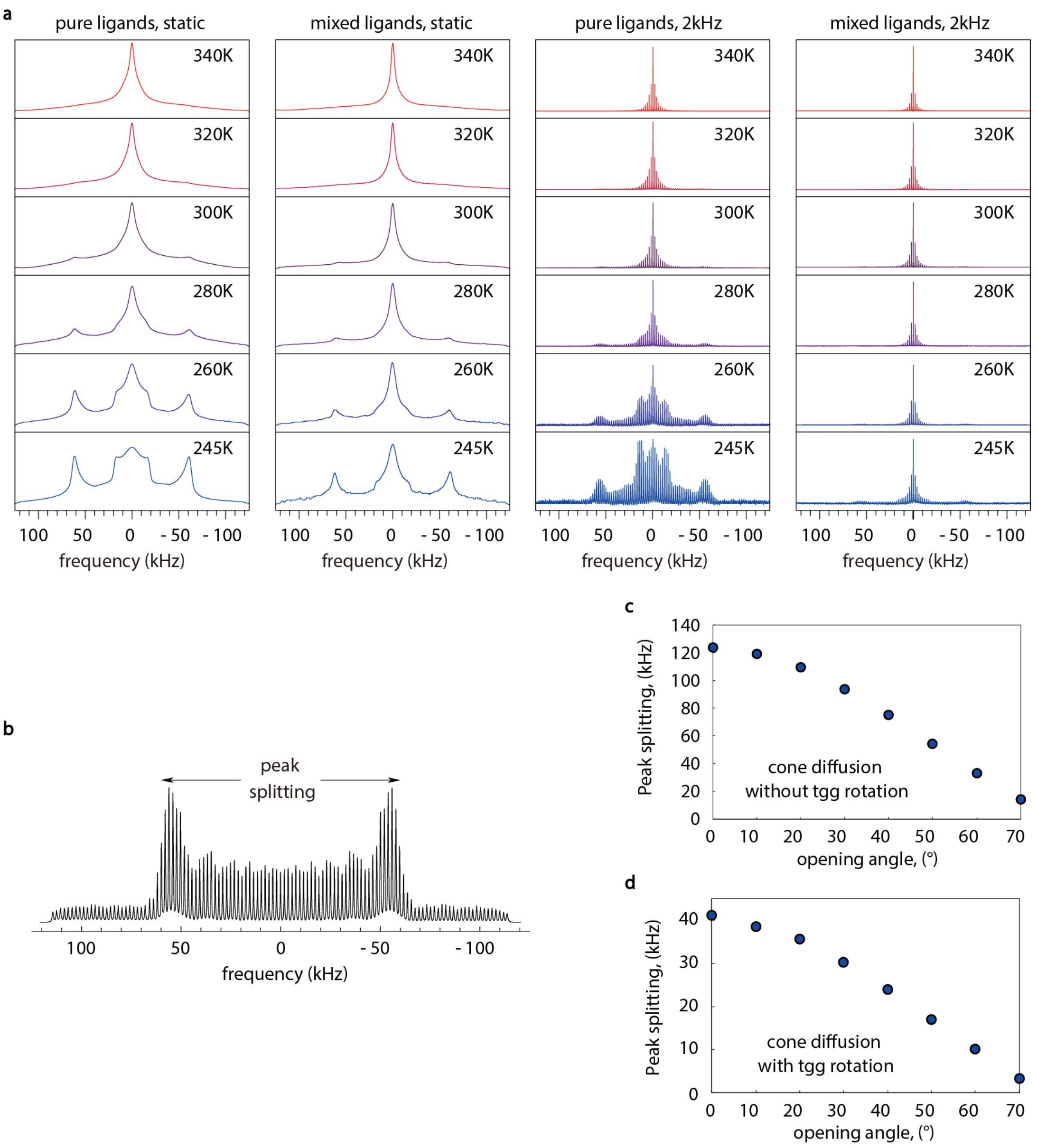

Supplementary Figure 3. Deuterium lineshapes and simulation details. a, ^{2}H NMR performed on samples with deuterated myristate ligands. Deuterium lineshapes from 245 K to 340 K in static condition for nanocrystal-ligands complexes with pure ligands and mixed ligands. Deuterium lineshapes from 245 K to 340 K under 2 kHz MAS for nanocrystal-ligands complexes with pure ligands and mixed ligands. **b,** A deuterium pattern illustrates the peak splitting of quadrupolar interaction. The splitting varies with the opening angle for **c,** a simple cone diffusion model without tgg rotation and **d,** superimposed cone diffusion model with tgg rotation. The superimposed cone diffusion suits the dynamic mode of flexible ligands on nanocrystals.

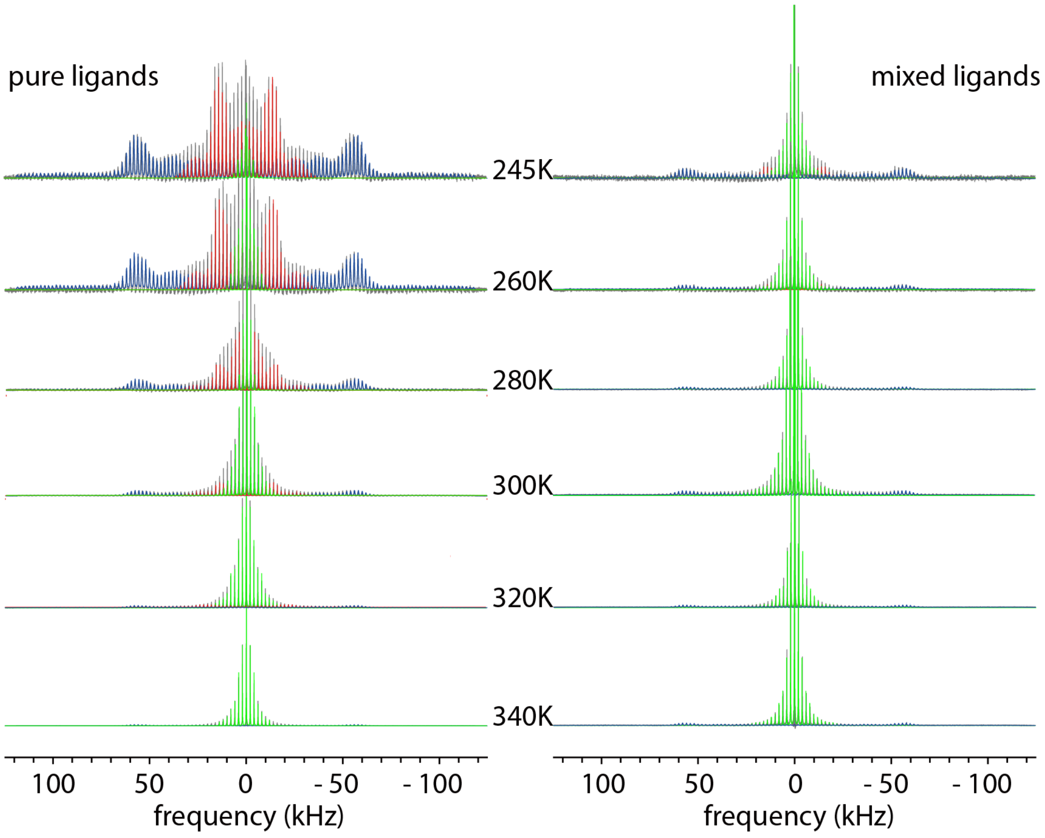

Supplementary Figure 4. Deconvolution results. The superpositions of static (blue), tgg (red) and cone diffusion (green) patterns for nanocrystal-ligands complexes with pure ligands or mixed ligands under 2 kHz MAS at variable temperatures. The grey patterns are the experimental results.

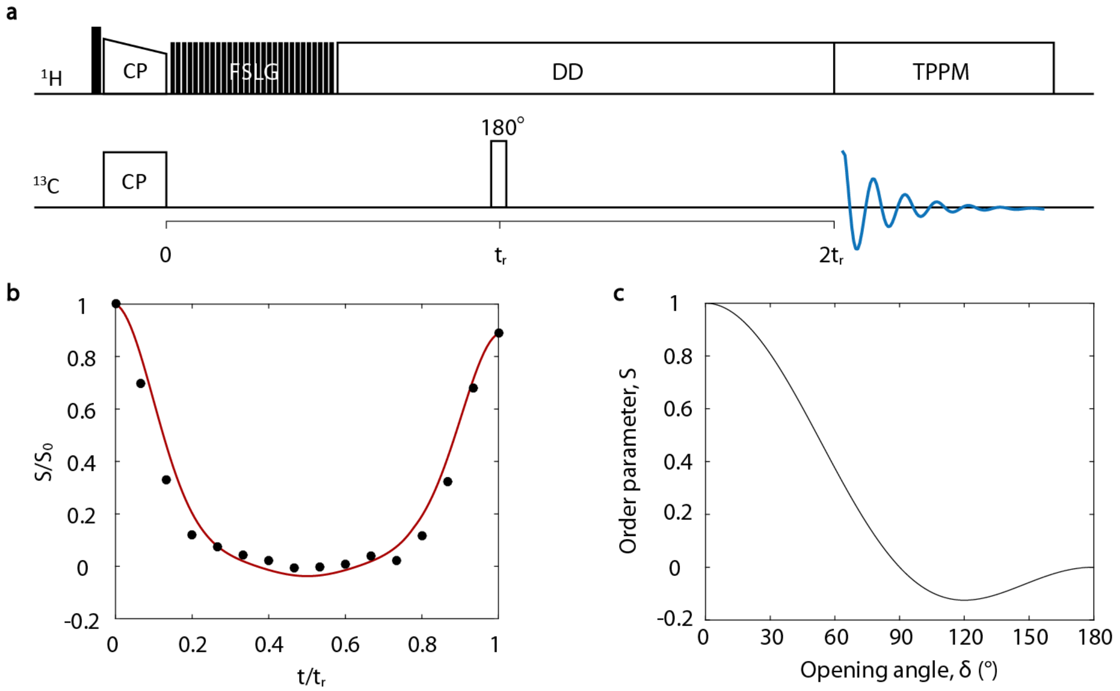

Supplementary Figure 5. DIPSHIFT details. a, DIPSHIFT sequence. The frequency-switched Lee-Goldburg (FSLG) pulses removes ^{1}H-^{1}H homonuclear coupling and the π-pulse between two rotor periods refocused ^{13}C CSA. Therefore, ^{13}C evolution with pure ^{1}H-^{13}C dipolar coupling is acquired during the FSLG period. **b,** DIPSHIFT results of methylene group of glycine. The red line is the simulated DIPSHIFT curve with a ^{1}H-^{13}C dipolar coupling of 19.1 kHz. **c,** The relation between opening angle and order parameter in DIPSHIFT experiments using the cone diffusion model.

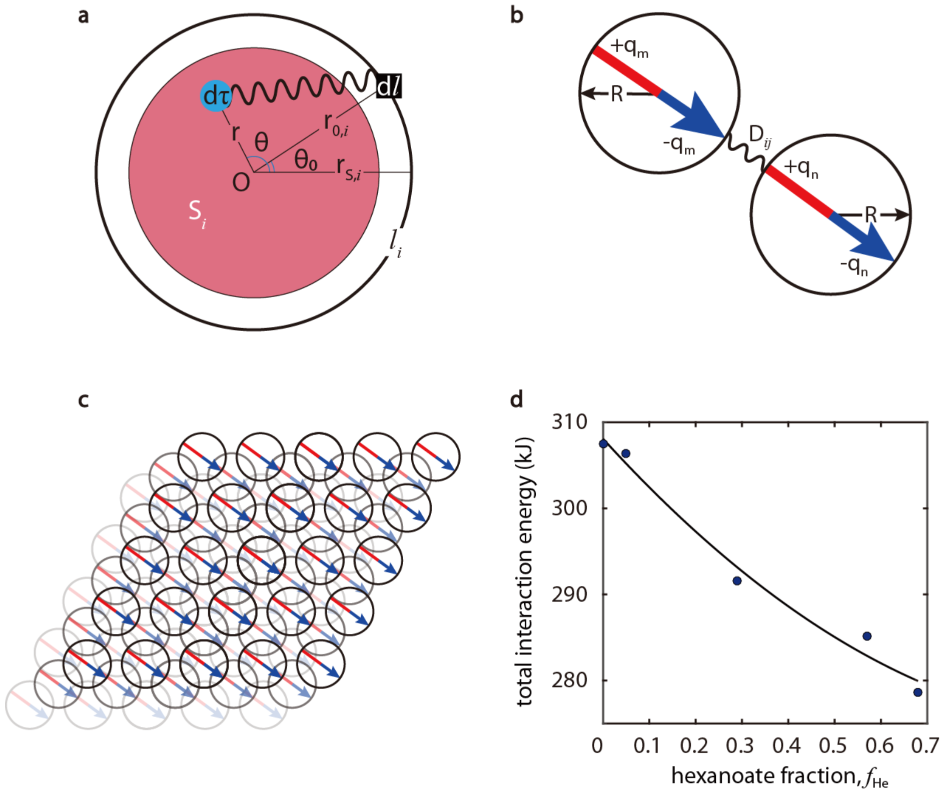

Supplementary Figure 6. Calculation of interaction energy between complexes. a, The graph illustrating the calculation of ligand-ligand interaction. The largest circle in black solid line indicates the circle potential. The blue ball indicates a CH_2 group which is confined in the free domain colored in pink. **b,** The graph illustrating the calculation of inter-particle interaction between two nanocrystals. **c,** The orientations of dipoles in a face-centered cubic lattice. **d,** The total interaction energy calculated at different hexanoate fractions. The solid line is the polynomial fitting curve described in the text above.

Supplementary Table 1. Elemental analysis results and calculated surface ligand density.

initial ratio (He:My)	hexanoate fraction (f_{He})	H wt%	C wt%	surface density
0:1	0	4.46	27.72	135
0.1:1	0.05	4.37	27.24	135
0.5:1	0.29	3.92	24.55	134
0.7:1	0.57	3.32	21.01	133
1:1	0.68	3.05	19.42	133
2:1	0.74	2.88	18.45	132
5:1	0.95	2.36	15.34	131

The hexanoate fractions were obtain from GC measurements. For different hexanoate fractions, the surface density of ligands on CdSe nanocrystals are about the same.

Supplementary Table 2. Averaged dipolar couplings and opening angles of methylene groups obtained from DIPSHIFT experiments.

f_{He}	Methylene position	2	3	5*	4-6	7-12	13	14
0	Averaged coupling, kHz	15	14	\	13.1	8	7.4	3.2
	Opening angle, °	32	36	\	39	57	59	75
0.05	Averaged coupling, kHz	15	14	\	13.1	7.5	7.2	3.2
	Opening angle, °	32	36	\	39	59	60	75
0.29	Averaged coupling, kHz	15	12.8	12.1	12.1	7.9	5.4	3.2
	Opening angle, °	32	40	43	43	57	66	75
0.57	Averaged coupling, kHz	15	12.6	11.9	11.9	7.7	5.3	3.1
	Opening angle, °	32	41	44	44	58	66	75
0.68	Averaged coupling, kHz	15	12	11.9	11.5	7.1	5	3
	Opening angle, °	32	43	44	45	60	68	75

* indicates the distinguishable No. 5 CH_2 group of hexanoate.

From No. 2 to No. 13 carbon units of myristates, the averaged ^1H-^{13}C couplings decrease and the opening angles increase. The signals of No. 4-6 and No. 7-12 carbon units overlap in the ^{13}C spectra. When hexanoate fraction increases, the flexibility increases for each carbon segment except the No. 2 unit which has an unchanged rigidity. The averaged coupling of the No. 14 unit is close to zero due to the fast rotation of methyl group.

Supplementary references

1 Pu, C. *et al.* Highly reactive, flexible yet green Se precursor for metal selenide nanocrystals: Se-octadecene suspension (Se-SUS). *Nano Res.* **6**, 652 (2013).

2 Zhou, J., Pu, C., Jiao, T., Hou, X. & Peng, X. A two-step synthetic strategy toward monodisperse colloidal CdSe and CdSe/CdS core/shell nanocrystals. *J. Am. Chem. Soc.* **138**, 6475 (2016).

3 Yang, Y., Li, J., Lin, L. & Peng, X. An efficient and surface-benign purification scheme for colloidal nanocrystals based on quantitative assessment. *Nano Res.* **8**, 3353 (2015).

4 Li, J., Chen, J., Shen, Y. & Peng, X. Extinction coefficient per CdE (E = Se or S) unit for zinc-blende CdE nanocrystals. *Nano Res.* (2018).

5 Yu, W. W., Qu, L., Guo, W. & Peng, X. Experimental determination of the extinction coefficient of CdTe, CdSe, and CdS nanocrystals. *Chem. Mater.* **15**, 2854-2860 (2003).

6 Yang, Y. *et al.* Entropic ligands for nanocrystals: from unexpected solution properties to outstanding processability. *Nano Lett.* **16**, 2133 (2016).

7 Yang, Y., Qin, H. & Peng, X. Intramolecular entropy and size-dependent solution properties of nanocrystal–ligands complexes. *Nano Lett.* **16**, 2127 (2016).

8 deAzevedo, E. R., Hu, W. G., Bonagamba, T. J. & Schmidt-Rohr, K. Centerband-only detection of exchange: efficient analysis of dynamics in solids by NMR. *J. Am. Chem. Soc.* **121**, 8411 (1999).

9 deAzevedo, E. R., Hu, W. G., Bonagamba, T. J. & Schmidt-Rohr, K. Principles of centerband-only detection of exchange in solid-state nuclear magnetic resonance, and extension to four-time centerband-only detection of exchange. *J. Chem. Phys.* **112**, 8988-9001 (2000).

10 Luo, W. & Hong, M. Determination of the oligomeric number and intermolecular distances of membrane protein assemblies by anisotropic ^{1}H-driven spin diffusion NMR spectroscopy. *J. Am. Chem. Soc.* **128**, 7242-7251 (2006).

11 Bain, A. D. Chemical exchange in NMR. *Prog. Nucl. Magn. Reson. Spectrosc.* **43**, 63-103 (2003).

12 Olender, Z. *et al.* Carbon-13 chemical-shift correlation, spin diffusion and self diffusion in

isotopically enriched tropolone. *J. Magn. Reson., Ser A* **120**, 31-45 (1996).

13 Vanderhart, D. L. Natural-abundance ^{13}C-^{13}C spin exchange in rigid crystalline organic solids. *J. Magn. Reson.* **72**, 13-47 (1987).

14 Girardeau, T. E., Leisen, J. & Beckham, H. W. Chain dynamics of poly(oxyethylene) in nanotubes of α-cyclodextrin by solid-state ^{2}H NMR. *Macromolecular Chemistry and Physics* **206**, 998-1005 (2005).

15 Yao, Y. F., Graf, R., Spiess, H. W. & Rastogi, S. Restricted segmental mobility can facilitate medium-range chain diffusion: a NMR study of morphological influence on chain dynamics of polyethylene. *Macromolecules* **41**, 2514-2519 (2008).

16 Vold, R. L. & Hoatson, G. L. Effects of jump dynamics on solid state nuclear magnetic resonance line shapes and spin relaxation times. *J. Magn. Reson.* **198**, 57 (2009).

17 Hong, M., Gross, J. D. & Griffin, R. G. Site-resolved determination of peptide torsion angle φ from the relative orientations of backbone N−H and C−H bonds by solid-state NMR. *J. Phys. Chem. B* **101**, 5869 (1997).

18 Goldburg, W. I. & Lee, M. Nuclear magnetic resonance line narrowing by a rotating rf field. *Phys. Rev. Lett.* **11**, 255-258 (1963).

19 Bielecki, A., Kolbert, A. C. & Levitt, M. H. Frequency-switched pulse sequences: Homonuclear decoupling and dilute spin NMR in solids. *Chem. Phys. Lett.* **155**, 341-346 (1989).

20 Levitt, M. H., Kolbert, A. C., Bielecki, A. & Ruben, D. J. High-resolution ^{1}H NMR in solids with frequency-switched multiple-pulse sequences. *Solid State Nucl. Magn. Reson.* **2**, 151-163 (1993).

21 Reichert, D. & Saalwächter, K. Dipolar coupling: molecular-level mobility. *eMagRes* (2008).

22 Lipari, G. & Szabo, A. Model-free approach to the interpretation of nuclear magnetic resonance relaxation in macromolecules. 1. Theory and range of validity. *J. Am. Chem. Soc.* **104**, 4546-4559 (1982).

23 Lipari, G. & Szabo, A. Model-free approach to the interpretation of nuclear magnetic resonance relaxation in macromolecules. 2. Analysis of experimental results. *J. Am. Chem. Soc.* **104**, 4559-4570 (1982).

24 Fedotov, V. D. & Schneider, H. *Structure and dynamics of bulk polymers by NMR-methods*. Vol. 21 (Springer, 1989).

25 Krushelnitsky, A. G., Hempel, G. & Reichert, D. Simultaneous processing of solid-state NMR relaxation and 1D-MAS exchange data: the backbone dynamics of free vs. binase-bound barstar. *Biochim. Biophys. Acta* **1650**, 117-127 (2003).

26 Israelachvili, J. N. in *Intermolecular and surface forces, Third Edition* (Academic Press, 2011).

27 Talapin, D. V., Shevchenko, E. V., Murray, C. B., Titov, A. V. & Král, P. Dipole−dipole interactions in nanoparticle superlattices. *Nano Lett.* **7**, 1213 (2007).
